# P27 Rethinking UTI diagnosis: how gold *is* the ‘gold standard’ and how much does antimicrobial susceptibility testing really matter?

**DOI:** 10.1093/jacamr/dlad077.031

**Published:** 2023-08-02

**Authors:** Joanna Diggle, Jeroen Nieuwland, Emma Hayhurst

**Affiliations:** Public Health Wales/University of South Wales, UK; University of South Wales/Llusern Scientific, UK; University of South Wales/Llusern Scientific, UK

## Abstract

**Objectives:**

To analyse the results provided by culture methods for UTI diagnosis and estimate its potential effectiveness for clinical decision making.

**Methods:**

The results of all 80981 urine samples processed by the Public Health Wales laboratory in Cardiff in 2022 were collated and analysed. Prevalence and resistance data were collected and analysed using Datastore. Specific aims were as follows: (i) determine the prevalence of ‘no growth’ and ‘mixed growth’ samples, and of each individual uropathogen; (ii) determine resistance rates for each pathogen; and (iii) estimate the potential effectiveness of these culture results for making clinical decisions.

**Results:**

Of the 80981 urine samples analysed, 22775 (28%) were negative following flow cytometry analysis and were not cultured. Of the 58206 urine samples cultured, 18656 (32%) showed no growth and 20516 (35%) showed mixed growth and were not analysed further. A total of 19034 (33%) showed growth of one or more specific pathogens. The most common uropathogen was *Escherichia coli* (58%), followed by *Klebsiella pneumoniae* (8.6%), *Enterococcus* spp. (7.3%) and *Proteus mirabilis* (4.2%). Resistance rates varied by pathogen. Resistance to nitrofurantoin in *E. coli* was low (2.4%) but resistance to trimethoprim amongst *E. coli* isolates was higher (30.5%). *Klebsiella*, *Pseudomonas* and *Proteus* spp. were not routinely tested for resistance to nitrofurantoin, presumably due to high levels of inherent resistance. Of the 22 *P. mirabilis* isolates tested, all were resistant to nitrofurantoin. Trimethoprim resistance was high for all species routinely tested. Only 24% of all samples analysed returned an antimicrobial susceptibility test (AST) result. AST results relevant to the first-choice antibiotics of nitrofurantoin or trimethoprim were obtained for only 16% and 19% of all urines tested, respectively. Overall resistance to nitrofurantoin was 2.8% and to trimethoprim 32%, representing 0.44% and 6% of total samples tested, respectively.

**Conclusions:**

These results highlight issues with laboratory culturing. Over one-third of all samples cultured did not return a usable clinical result due to mixed growth and AST results were returned for less than one-quarter of all samples tested. The clinical utility of AST depends on the specific antibiotic and species being tested. With nitrofurantoin resistance currently so low in all species routinely tested, knowing whether there is an infection and which bacteria is causing it will have more impact than performing susceptibility testing for this antibiotic. However, for trimethoprim, resistance rates are higher and more variable, so AST has more potential clinical impact.

